# Sliding friction of graphene/hexagonal –boron nitride heterojunctions: a route to robust superlubricity

**DOI:** 10.1038/s41598-017-10522-8

**Published:** 2017-09-07

**Authors:** D. Mandelli, I. Leven, O. Hod, M. Urbakh

**Affiliations:** School of Chemistry and The Sackler Center for Computational Molecular and Materials Science, Tel Aviv University, 6997801 Tel Aviv, Israel

## Abstract

The origin of ultra-low friction exhibited by heterogeneous junctions of graphene and hexagonal boron nitride (*h*-BN) is revealed. For aligned interfaces, we identify a characteristic contact size, below which the junction behaves like its homogeneous counterparts with friction forces that grow linearly with the contact area. Superlubricity sets in due to the progressive appearance of Moiré patterns resulting in a collective stick-slip motion of the elevated super-structure ridges that turns into smooth soliton-like gliding with increasing contact size. Incommensurability effects are enhanced in misaligned contacts, where the friction coefficients further drop by orders of magnitude. Our fully atomistic simulations show that the superlubric regime in graphene/*h*-BN heterostructures persists up to significantly higher loads compared to the well-studied twisted homogeneous graphene interface. This indicates the potential of achieving robust superlubricity in practical applications using two-dimensional layered materials heterojunctions.

## Introduction

The high surface-to-volume ratio of micro- and nano-mechanical contacts greatly enhances the undesired effects of wear and frictional energy dissipation. Hence, considerations of friction reduction and protection of moving components have a central role in the design of miniaturized devices^1^. Further challenges are posed by the inadequacy of traditional lubrication methods, such as liquid-phase lubricants, which cease to be effective in extremely confined conditions and under high loads^2^.

Surprisingly, solid/solid crystalline interfaces offer a unique way to reduce friction. Unlike standard lubricants, solid lubrication arises from incommensurability of rigid interfaces causing effective cancellation of interfacial interactions^3^. This, in turn, reduces sliding energy barriers resulting in practically frictionless motion - a phenomenon referred to as structural superlubricity^4^.

In order to achieve a superlubric state the junction should be constructed from clean, chemically inert, and atomically flat rigid surfaces. These requirements are naturally met by hexagonal layered materials such as graphite, hexagonal boron nitride (*h*-BN), and members of the metal dichalcogenides family including *2H*-molybdenum disulfide (MoS_2_) and tungsten disulfide (WS_2_)^5^. Their anisotropic structure, consisting of strong covalent intralayer bonding and weaker interlayer dispersive interactions, allows for efficient interlayer sliding and resistance to wear. Superlubricity in hexagonal layered materials was first demonstrated for the homogeneous junction of a nanoscale multilayer graphene flake attached to a friction force microscope tip sliding over a flat graphite surface^6, 7^. A direct correlation between interfacial lattice commensurability and sliding friction was clearly demonstrated. There, commensurate junctions exhibited high friction stick-slip motion rapidly switching into a superlubric state with increasing interlayer misfit angle. Recently, similar phenomena have been observed at microscale graphitic junctions^8–10^.

Despite the great promise for using graphitic junctions in solid lubrication, there remain two main drawbacks that may hinder superlubricity. First, contacts between identical materials may exhibit spontaneous dynamic reorientation that locks the system into a high friction commensurate state^11^. Furthermore, superlubricity may be eliminated with increasing load due to load-induced commensuration^12^, edge effects^13^, and elasticity induced local commensurability^14, 15^.

To overcome these problems heterojunctions between different layered materials may offer an accessible alternative^16–19^. The intrinsic intralayer lattice constant mismatch between adjacent layers prohibits the formation of commensurate contacts when their size approaches the periodicity of the corresponding Moiré superstructures^20^. In particular, the structural similarity between graphene and its inorganic *h*-BN counterpart stimulated studies on the mechanical and frictional properties of their planar heterojunctions^21, 22^. For flat rigid graphene/*h*-BN interfaces registry considerations predicted robust superlubricity with weak dependence on the interlayer lattice orientation^16^. This analysis, however, ignored important effects such as layer elasticity, out-of-plane atomic displacements, and energy dissipation that may considerably influence the frictional response of the system.

To take these effects into account, we perform fully atomistic quasi-static molecular dynamics simulations of graphene flakes sliding over rigid *h*-BN substrates mimicking typical friction force microscopy experiments^7^. The effects of important factors including interlayer alignment, contact size, and external load are studied. For aligned lattices we find a non-monotonic friction force dependence on the contact size that originates from the progressive appearance of the Moiré pattern. The latter defines a characteristic contact size, above which structural superlubricity sets in. In misaligned flakes, friction is found to be orders of magnitudes smaller with no significant size dependence. Unlike the homogeneous graphitic junction, our simulations show that the superlubric state appears even for the aligned graphene/*h*-BN contact, which indicates the possibility to eliminate the undesired effects of self-reorientation processes. Moreover, superlubricity endures up to significantly larger loads for rotated heterogeneous interfaces. These findings are promising in view of achieving robust superlubricity in many practical applications.

## Model

**Figure 1:**
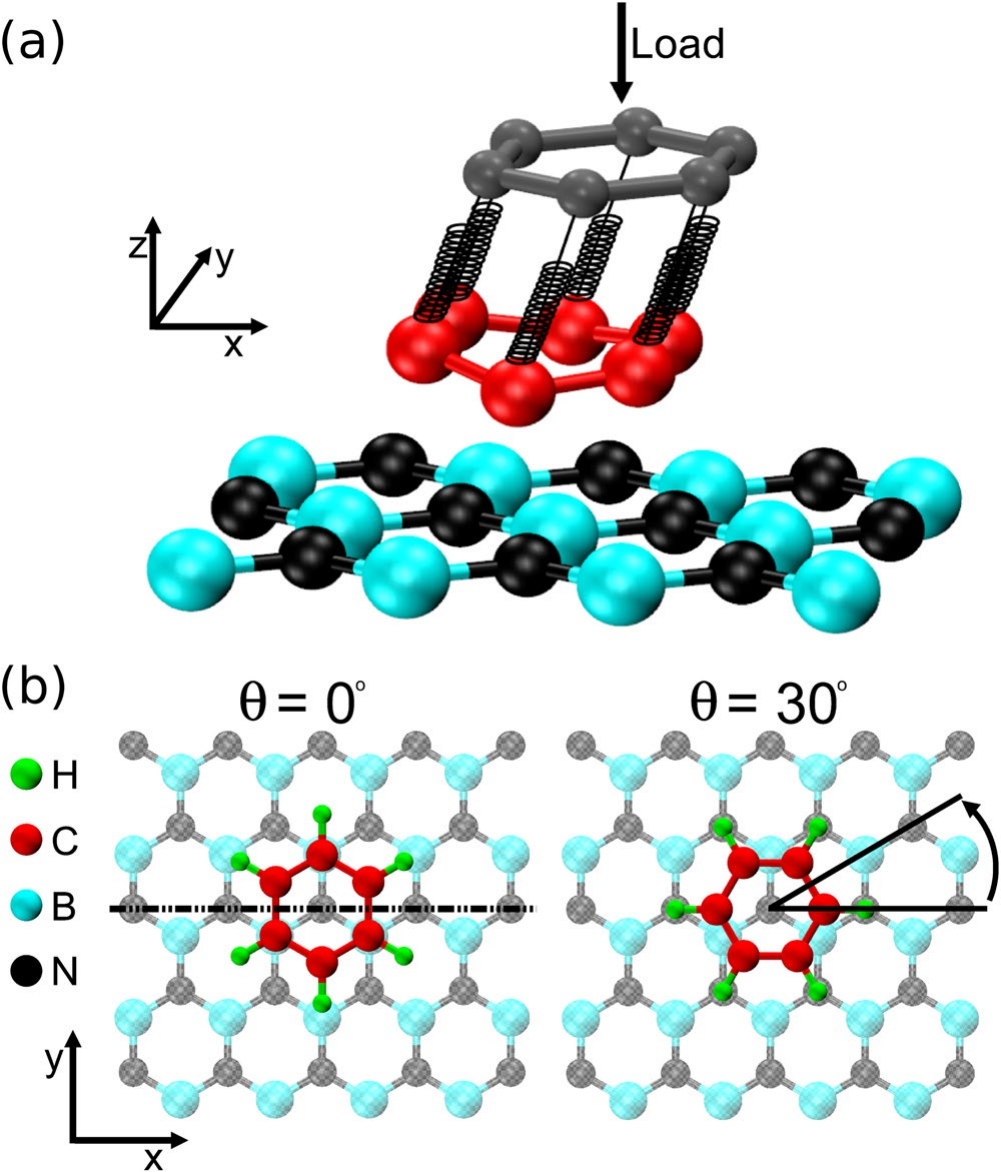
Schematics of the simulation setup of a friction force microscopy experiment. (**a**) A schematic representation of the simulation protocol: a rigid tip (dark-grey spheres) is moved parallel to a flat, rigid, substrate monolayer (light blue and black spheres). The sliding flake (red spheres) is dragged by springs connecting each atom to the corresponding image-atom of the tip model. The normal load is modeled by a homogeneous force acting on the tip. (**b**) Examples of the heterogeneous interfaces used in the simulations. The initial positions at the two angular orientations considered are shown. The dot-dashed line indicates the scan direction during sliding. For clarity, the flake and duplicate are represented by a benzene molecule. The actual hexagonal flake sizes used in the simulations are explicitly noted throughout the text.

Our simulation setup, aiming to mimic friction force microscopy (FFM) experiments, is schematically presented in Fig. 1a. We consider a graphene flake dragged along a rigid graphene or *h*-BN substrate. The pulling tip is represented by a rigid duplicate of the dragged flake, whose center of mass is driven parallel to the hexagonal substrate along its zigzag direction (x-direction in our chosen reference frame, see Fig. 1b). The duplicate interacts with the dragged flake via a set of harmonic springs connecting each image tip atom to its counterpart on the flake. The interaction between the flake and the underlying surface is modeled by dedicated interlayer potentials (see Methods section for further details). Normal loads are modeled by applying a constant force at the center of mass of the rigid duplicate, in a direction perpendicular to the substrate. Our quantity of interest is the instantaneous friction force *F*_*x*_, defined as the total force experienced by the tip in the direction of sliding:

1 $${F}_{x}=\sum _{i=1}^{{N}_{at}}{K}_{||}\,({x}_{i}^{tip}-{x}_{i}^{flake})$$

Here, ${N}_{at}$ is the total number of atoms in the flake, *K*_*||*_ is the lateral spring constant, and ${x}_{i}^{tip}$, ${x}_{i}^{flake}$ are the positions of the *i*-th atom in the rigid duplicate and in the flake, respectively. In our quasi-static protocol (see Methods section) *F*_*x*_ is a periodic function of the tip position. This allows us to define the static and kinetic friction forces as the maximal and average force values along one period of the friction trace, respectively. The corresponding friction coefficients are evaluated via a numerical derivative of the friction force with respect to the applied load (see Methods section).

## Simulation Results

### Aligned contacts

**Figure 2:**
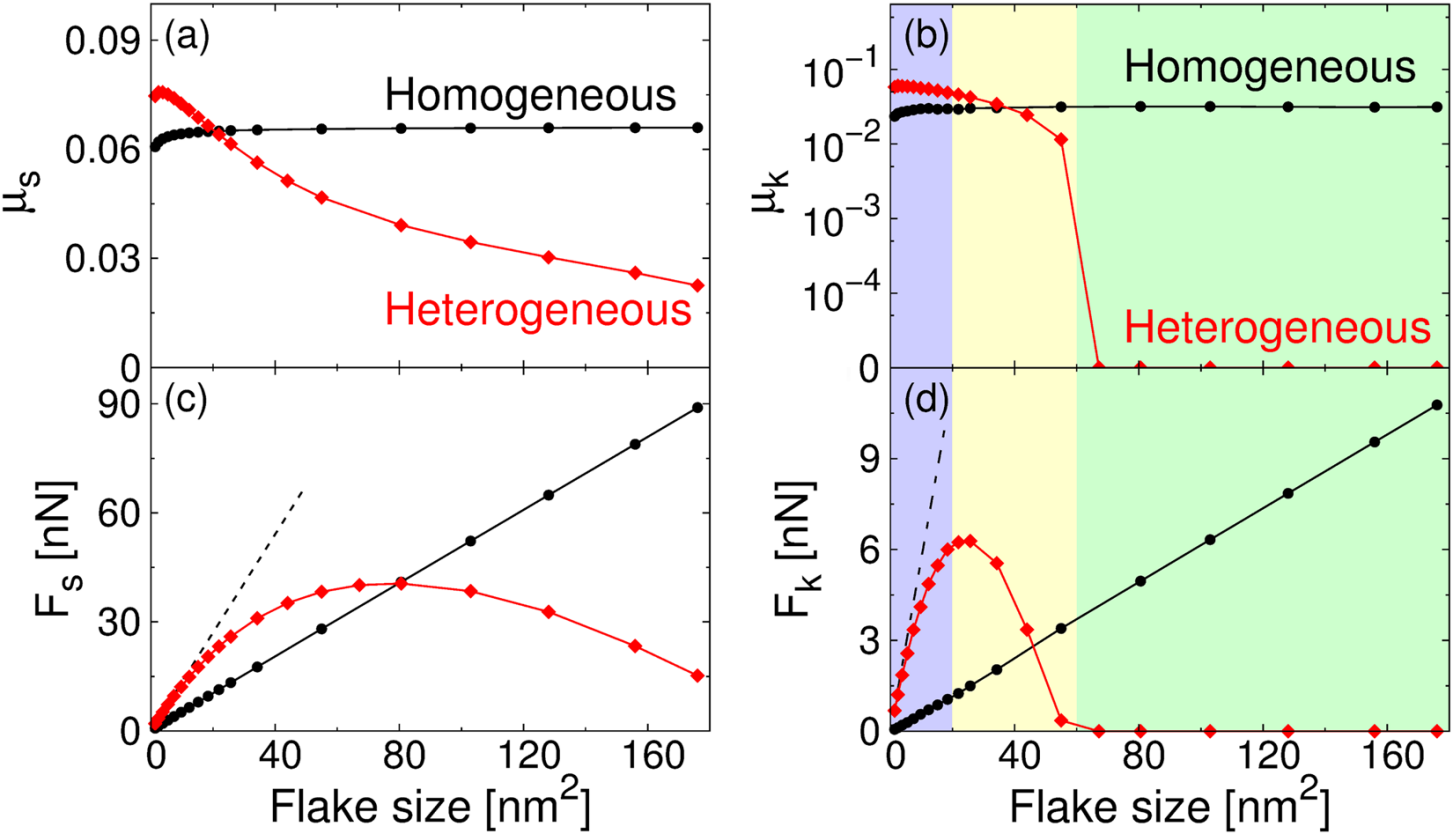
Contact size dependence of friction calculated for the aligned (θ = 0°) homogeneous and heterogeneous interfaces. (**a**),(**b**) Static and kinetic friction coefficients as a function of the flake size for the homogeneous (black) and heterogeneous (red) junctions. (**c**) The corresponding static and (**d**) kinetic friction forces calculated at a normal load of L = 0.1 nN/atom. The dashed lines show the size dependence of the friction forces obtained considering a fictitious commensurate graphene/*h*-BN interface at the lattice constant of *h*-BN. Lines connecting dots are provided as guides to the eye. Similar trends are observed for all values of the load investigated as reported in section S1 of the Supplementary Information. The purple, yellow, and green regions appearing in panels (**b**) and (**d**) mark the coherent stick-slip, the collective stick-slip, and the soliton-like smooth sliding regimes, respectively, as discussed in the main text.

We first consider the aligned case (θ = 0°, see left panel of Fig. 1b), where the lattice vectors of the tip and the substrate are kept parallel. Figure 2a and b report the static and kinetic friction coefficients as a function of the flake size, respectively. In the homogeneous graphene/graphene junction both static and kinetic friction coefficients show an initial small increase that saturates above a size of ~20 nm^2^ (black lines). This behavior can be attributed to local flake/substrate lattice incommensuration appearing at the flake edges due to reduced carbon-carbon bond-lengths. As the flake size increases the contribution of edge atoms reduces and the static and kinetic friction coefficients saturate at μ_s_ = 0.07 and μ_k_ = 0.03, respectively, which compare well with previous theoretical investigations^13^ and experimental results^23^. The corresponding friction forces present a linear increase (see black lines in Fig. 2c,d, respectively) as expected in commensurate contacts.

**Figure 3:**
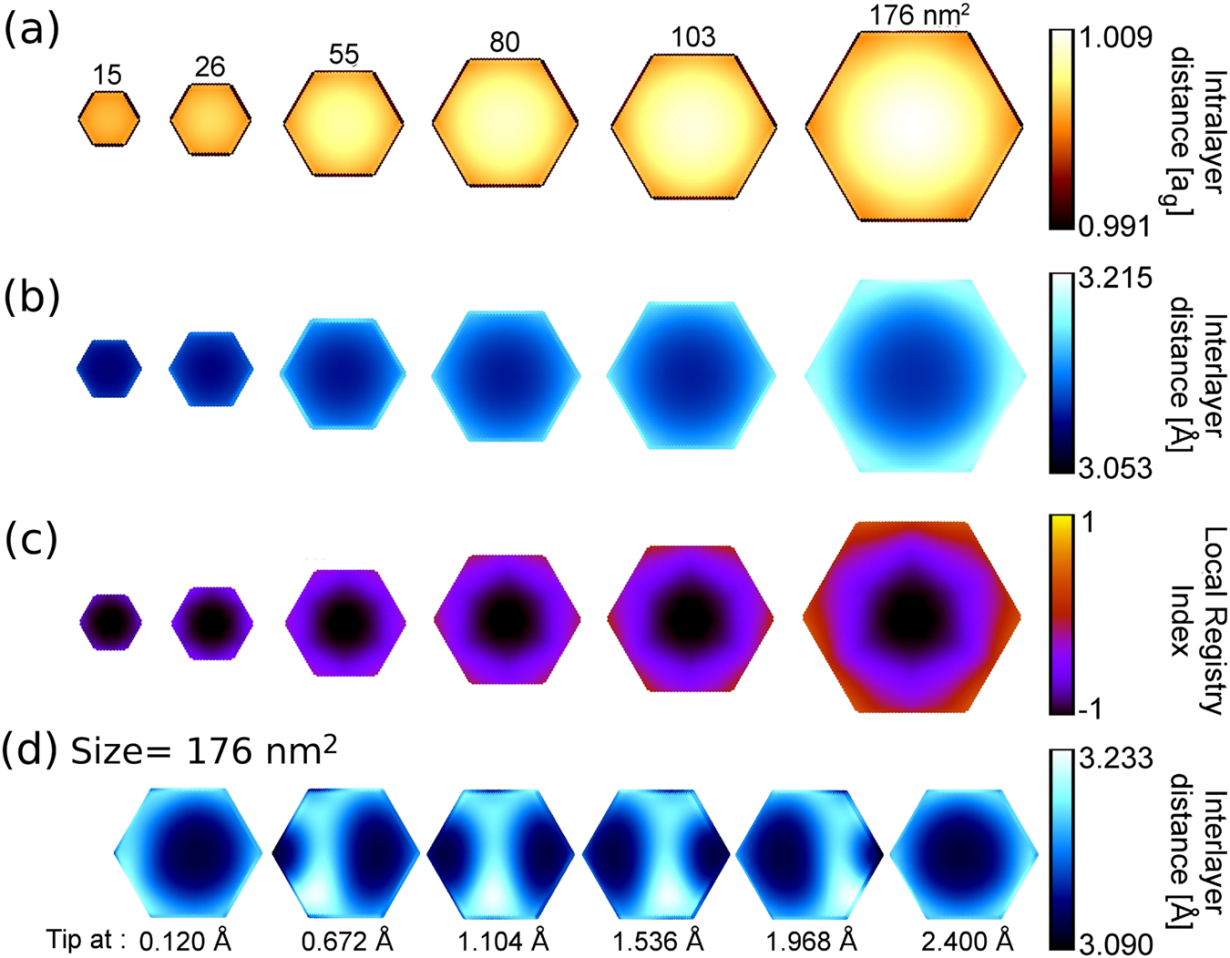
Emergence  of the Moiré superstructure with increasing graphene/*h*-BN heterojunction contact size. Panels (**a**), (**b**), and (**c**) present the structures of hexagonal graphene flakes relaxed over a rigid *h*-BN substrate as function of contact size. The color maps of panels (**a**) and (**b**) show the normalized intralayer C-C bond length and the interlayer distance, respectively. Panel (**c**) reports color maps of the local registry index defined in ref. 26. Here dark regions correspond to the energetically favorable C stacking mode and bright regions correspond to the energetically unfavorable A stacking mode of the heterojunction, clearly indicating the appearance of the Moiré superstructure with increasing contact area. Panel (**d**) demonstrates the soliton propagation sliding mechanism during the lateral motion of the 176 nm^2^ flake. All results reported in this figure were obtained under a normal load of 0.1 nN/atom.

A completely different behavior is observed for the aligned heterogeneous *h*-BN/graphene contact, where the static and kinetic friction forces exhibit a non-monotonic dependence on the flake size with a corresponding reduction of the friction coefficients (see red lines in Fig. 2, and section S1 in the Supplementary Information for results at different normal loads). The origin of this curious behavior can be traced to the Moiré superstructure appearing at the heterogeneous interface due to the intralayer lattice constant mismatch of graphene and *h*-BN^20, 24, 25^. These structures are characterized by extended nearly-commensurate regions, separated by domain walls. Within the former, the two surfaces remain at their optimal interlayer distance while their hexagonal lattices stretch or compress to adapt to each other (see Fig. 3a,b). The resulting tension is compensated by forming elevated ridges characterized by out-of-plane deformation and loss of lattice commensurability (see Fig. 3b).

At flake sizes considerably smaller than the Moiré super-cell the system is practically commensurate, and the static friction force grows linearly with the contact size, following quantitatively the ideal rise estimated using a fictitious perfectly commensurate graphene/*h*-BN interface (see dashed line in Fig. 2c). This results from the fact that all flake atoms coherently cross similar potential energy barriers. When the flake size accommodates the entire nearly-commensurate region (see the local registry index^26^ maps of Fig. 3c) the static friction force obtains its maximal value. With further increase of flake size, the appearance of the elevated ridges results in gradual reduction of the static friction force. This can be attributed to the incommensurability of the ridge regions and to the fact that their appearance induces a lift-up of the entire flake. We therefore find that the variation of the static friction force with the flake size is determined by the intrinsic properties of the contact and is independent of the driving apparatus (see section S2 in the Supplementary Information).

The kinetic friction force exhibits three distinct frictional regimes: (i) At small sizes (below 20 nm^2^) the kinetic friction force grows linearly with the contact size; (ii) A cross-over regime, characterized by deviation from linearity followed by a significant reduction of the kinetic friction force, is observed for intermediate flake sizes (20–60 nm^2^); (iii) Above a flake size of ~60 nm^2^ the kinetic friction force nearly vanishes.

The characteristic length-scale controlling the transition between the first and second regime is the width of the elevated ridges, which depends on the intrinsic lattice mismatch and interlayer potential as well as on the externally applied load. When the flake diameter is considerably smaller than the width of these ridges (in our case 5 nm, roughly corresponding to a flake size of 30 nm^2^) the heterogeneous *h*-BN/graphene junction behaves as its homogeneous graphene/graphene counterpart. Here, the flake atoms move coherently across the potential energy landscape exhibiting stick-slip motion (see movie 1 in the Supplementary Information) that is well described by the standard Prandtl-Tomlinson model^27^. This explains the initial linear increase of the kinetic friction force observed up to a flake size of ~20 nm^2^ that, similar to the static friction force, follows quantitatively the fictitious perfectly commensurate graphene/*h*-BN interface behavior (dashed line in Fig. 2d).

For flake sizes comparable to the width of the domain walls the atoms movement loses coherence and the stick-slip motion is governed by soliton-like propagation of the ridges across the flake (see movie 2 in the Supplementary Information, demonstrating the stick-slip motion of the soliton). Since, now, only a fraction of the flake atoms needs to cross the potential energy barriers simultaneously this soliton motion results in an increased overshoot (a situation, where the flake surpasses the tip) of the entire flake during the slip event (see section S2 in Supplementary Information). This, in turn, leads to a momentary negative dragging force that reduces the overall kinetic friction compared to that of the commensurate junction (Fig. 2d).

Further increase of the flake size (above 60 nm^2^ in Fig. 2) results in a transition to smooth soliton sliding (see Fig. 3d, and the corresponding movie 3 in the Supplementary Information). This occurs when the stiffness of the driving apparatus, ${N}_{at}{K}_{||}$, becomes considerably larger than the steepness of the sliding energy potential that can be estimated as ${{\rm{F}}}_{{\rm{s}}}/{a}_{h-{\rm{BN}}}$. Here, ${a}_{h-{\rm{BN}}}$ is the periodicity of the *h*-BN lattice along the scan-line and ${{\rm{F}}}_{{\rm{s}}}$ is the size-dependent static friction force. Following the Prandtl-Tomlinson model the transition from stick-slip motion to smooth sliding occurs when the dimensionless parameter $\eta =2\pi {F}_{s}/({N}_{totat}{K}_{\parallel }{a}_{h-BN})$ exceeds a critical value^28^. At this point the mechanical instability resulting from the interplay between the external driving force and the opposing frictional force, exerted by the potential energy landscape, is eliminated. For the case of a single particle sliding atop a one-dimensional sinusoidal potential the critical transition value is $\eta =1$. In our setup, which includes a large number of interacting atoms sliding atop a complex three-dimensional potential energy landscape, the corresponding critical value becomes system dependent but remains close to unity for all conditions considered herein (see section S3 in the Supplementary Information).

In this superlubric regime the kinetic friction coefficient drops to vanishingly small values^29^ (see Fig. 2b) and the static friction coefficient exhibits a two-fold reduction (see Fig. 2a). Notably, for the largest flake, whose diameter is close to one Moiré periodicity, a full soliton sweep across the entire length of the flake corresponds to a nominal displacement by one lattice constant (see Fig. 3d). We note that in the present quasi-static treatment dynamic frictional effects are neglected. The latter may introduce additional contributions to energy dissipation thus somewhat enhancing the kinetic friction.

Therefore, the non-monotonic behavior of the kinetic friction force with contact size corresponds to a transition from coherent stick-slip motion of the entire flake lattice to a superstructure soliton propagation mechanism followed by a Tomlinson-like crossover to smooth sliding. Here the position of the kinetic friction force peak depends on the spring constant of the driving apparatus via an interplay between the increase of static friction with flake size and the overshoot of the friction force following the slip event (see section S2 in the Supplementary Information for further details).

**Figure 4:**
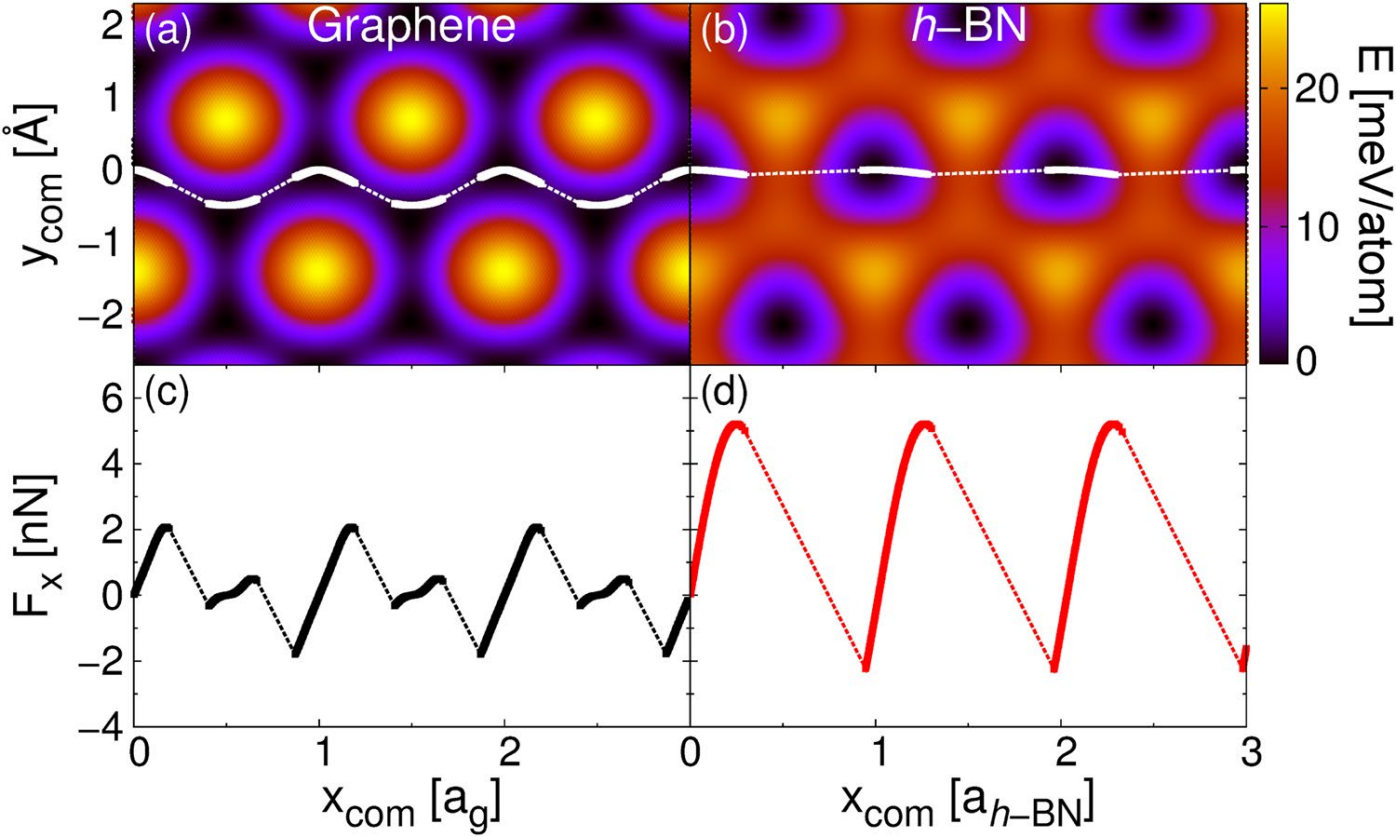
Comparison between the stick-slip motion of a small graphene flake sliding atop graphene and *h*-BN. Upper panels show the center-of-mass trajectories of the 4 nm^2^ hexagonal graphene flake sliding atop graphene (**a**) and *h*-BN (**b**). Bottom panels (**c**),(**d**) report the corresponding spring force traces. Simulations were performed under an external load of 0.1 nN/atom.The color maps in the upper panels show the two-dimensional sliding PES for a flat, rigid, crystalline flake of the same size, computed at a fixed interlayer distance equal to the average value measured in the corresponding quasi-static simulations. Since the maximal variations in the interlayer distance along the quasistatic trajectories are of 0.05 Å for the heterogeneous junction and 0.01 Å for the homogeneous junction these color maps represent well the actual sliding energy landscape.

We note that at small contact sizes both static and kinetic friction are larger in the heterojunction with respect to the homogeneous interface. This can be explained by examining the motion of the flake’s center-of-mass (C.O.M.). Figure 4a and b present the C.O.M. trajectories of a 4 nm^2^ graphene flake superimposed on the sliding potential energy surfaces (PESs) of the corresponding rigid graphene flake on rigid graphene and *h*-BN substrates, respectively. Naively, one could expect that the higher corrugation exhibited by the homogeneous junction would result in increased frictional forces. Nevertheless, atop the graphene substrate the graphene flake exhibits a zigzag motion (see Fig. 4a) avoiding the global PES maxima thus effectively crossing lower barriers along the sliding path, leading to a reduced stick-slip amplitude (Fig. 4c). The surface energy profile on *h*-BN does not exhibit such a smooth minimum energy path thus forcing the flake to cross a relatively high saddle-point (see Fig. 4b) resulting in pronounced stick-slip motion and increased friction (Fig. 4d). This effect becomes insignificant for larger flakes, where the soliton motion dominates the frictional behavior.

### Misaligned contacts

Let us now turn to discuss the rotated interfaces with a misfit angle of θ = 30°. In this case, for small loads both static and kinetic friction display insignificant dependence on the contact size (see section S4 in the Supplementary Information) in agreement with previous studies employing geometric considerations^30^. We therefore present here results for a fixed contact size of 2.4 nm^2^. For small normal loads both misaligned (homogeneous and heterogeneous) junctions are expected to display smooth-sliding. Nevertheless, above a given (system dependent) load, superlubricity should be eliminated.

**Figure 5:**
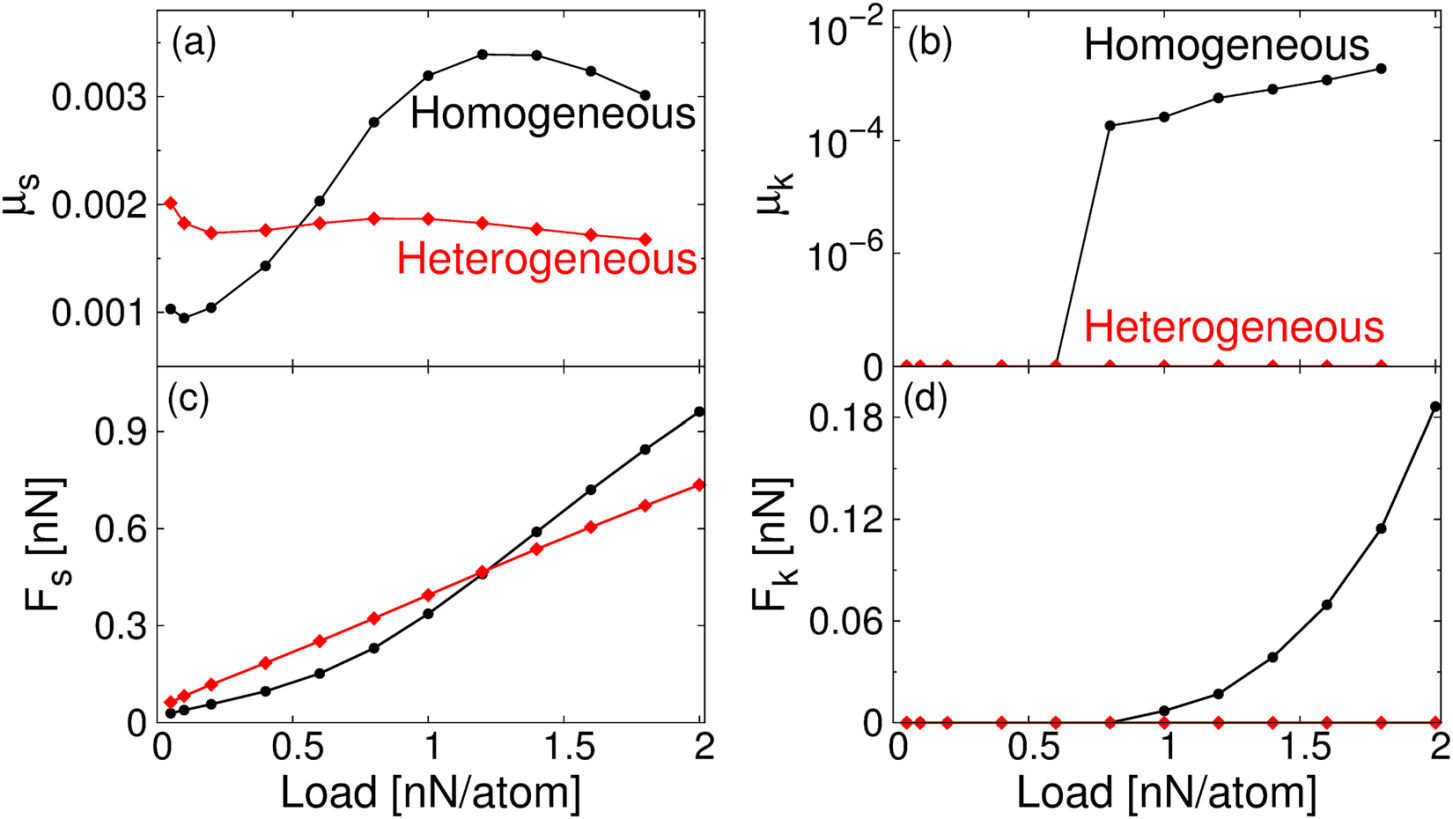
Load dependence of friction calculated for the misaligned (θ = 30°) homogeneous and heterogeneous interfaces. (**a**),(**b**) Static and kinetic friction coefficients as a function of the applied normal load for a flake size of 2.4 nm^2^. (**c**),(**d**) The corresponding static and kinetic friction forces. Lines connecting dots are provided as  guides to the eye.

To demonstrate this, we compare in Fig. 5 the load dependence of the static and kinetic friction coefficients and forces of the homogeneous and heterogeneous contacts. The static friction coefficient of the heterogeneous junction remains mostly constant throughout the external loads range considered, while that of the homogeneous junction exhibits a 3-fold increase with increasing load (Fig. 5a). Correspondingly, the static friction force of the former presents an Amonton-like behavior, growing linearly with the external normal load (Fig. 5c), while the latter slightly deviates from linearity.

The kinetic friction coefficient of both the homogeneous and heterogeneous misaligned junctions are found to be extremely small (in fact, below our numerical accuracy) at small loads (Fig. 5b). However, above a certain threshold load (~0.8 nN/atom in the present case) the friction coefficient of the homogeneous contact increases abruptly to values of µ_k_ ~10^−4^–10^−3^ (still within the superlubric regime), whereas that of the heterojunction remains negligible. In agreement with ref. 13, the kinetic friction force of the homogeneous interface is vanishingly small up to a normal load of ~0.8 nN/atom, above which it rapidly increases (see Fig. 5d). Remarkably, in the heterogeneous junction the kinetic friction force remains negligible (3–4 orders of magnitude smaller than the values observed in the corresponding aligned case) up to the largest load considered^31^.

**Figure 6:**
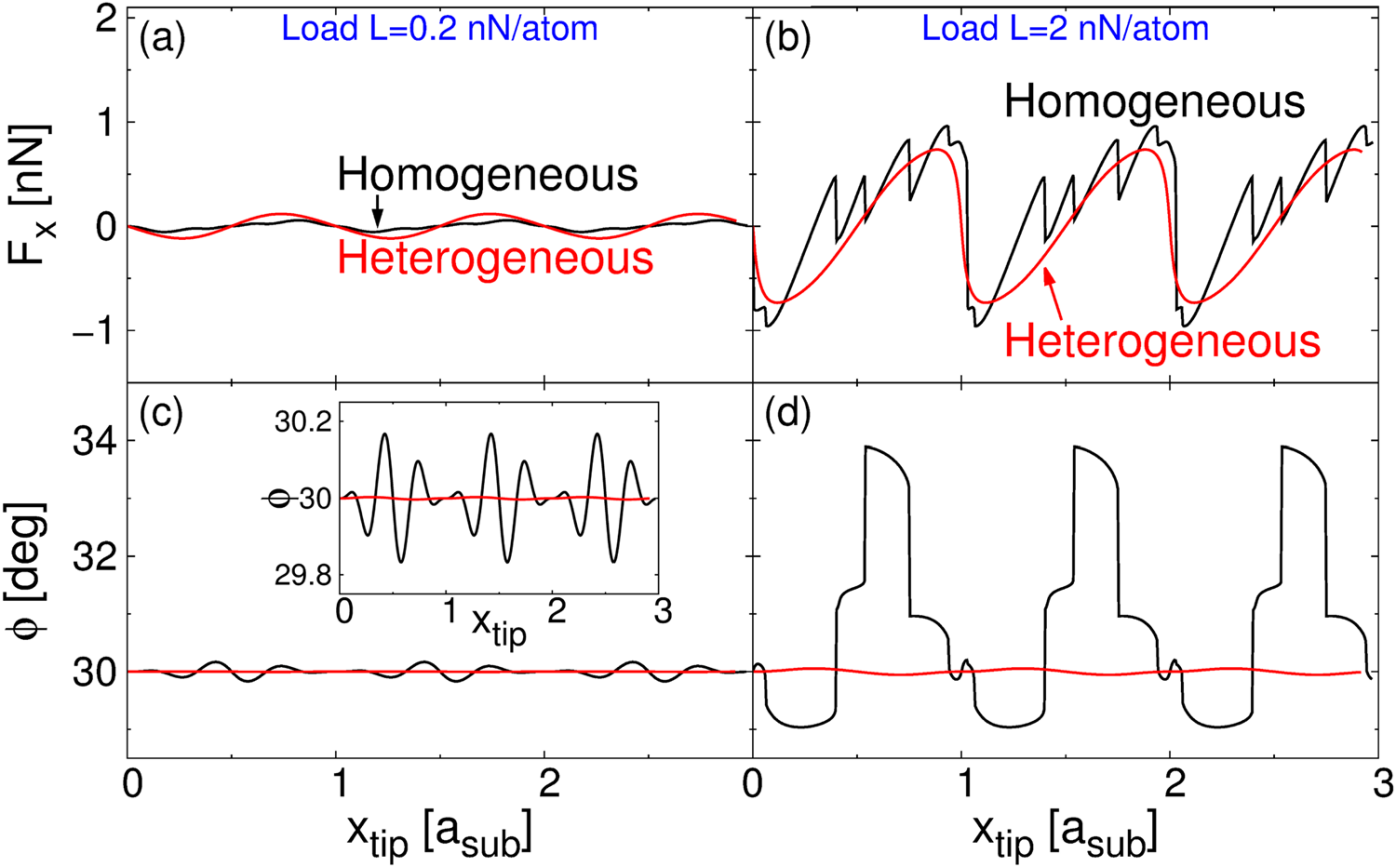
The effect of load on the spring force traces and angular flake trajectories of the rotated (θ = 30°) homogeneous and heterogeneous contacts. Panels (**a**) and (**b**) show the force traces of the 2.4 nm^2^ homogeneous (black lines) and heterogeneous (red lines) contacts obtained at normal loads of 0.2 nN/atom and 2.0 nN/atoms, respectively. Panels (**c**) and (**d**) show the corresponding global angular orientation of the flake along the trajectory. The inset in panel (**c**) is a zoom-in showing how, even at small loads, the orientation of the homogeneous junction undergoes larger fluctuations than its heterogeneous counterpart.

To rationalize this notable difference in kinetic friction behavior between the homogeneous and heterogeneous rotated interfaces we plot in Fig. 6 the driving spring force traces and the flake trajectories during the sliding motion. At small loads both systems display small lateral forces that average out throughout each period (Fig. 6a). Correspondingly, the flakes show very little angular deviations from the 30° misalignment imposed by the tip (Fig. 6c). Above a load of 0.8 nN/atom the homogeneous system exhibits erratic motion characterized by sudden angular reorientations^32^ (see black lines in Fig. 6d) accompanied by significant displacements in the direction perpendicular to the scan line (not shown). While at such loads the forces that the heterojunction exerts on the driving spring increases as well (see red line in Fig. 6b) the translational and rotational motion of the flake remain smooth (see red line in Fig. 6d). The origin of this erratic motion exhibited by the homogeneous contact was identified in ref. 13. to be related to pinning of the edge atoms to the underlying surface at high loads. This phenomenon is suppressed in the case of the heterogeneous junction due to the smoother sliding energy landscape experienced by the edge atoms. In fact, we found that at high loads (2 nN/atom), i.e. small interlayer distances, the overall corrugation of the sliding potential energy landscape of a single carbon atom is reduced by a factor of two for the case of a *h*-BN substrate, with respect to the case of a graphene substrate. Importantly, these edge effects become negligible in large flakes (size >10 nm^2^), where superlubricity is found to survive up to high loads also in the homogeneous contact (not shown).

## Conclusions

In this study, we presented a comparative molecular mechanics study of the frictional properties of homogeneous graphene/graphene and heterogeneous graphene/*h*-BN nanoscale contacts. We find that, for all contact sizes considered, the commensurate homogeneous interface exhibits highly dissipative stick-slip motion resulting in size-independent static and kinetic friction coefficients. In contrast, the corresponding heterogeneous junction exhibits a transition from stick-slip motion to smooth sliding as a function of contact size. This is attributed to the appearance of the Moiré superstructure resulting from the intrinsic lattice parameter misfit between the two surfaces. For small contact areas, the heterogeneous junction behaves like its homogeneous counterparts exhibiting coherent atomic motion with stick-slip characteristics. For contact sizes comparable to the width of the Moiré domain walls the flake’s motion occurs via soliton propagation of the elevated ridges. This results in dramatic reduction of the friction coefficient leading, eventually, to the onset of superlubricity even in the aligned contact thus suggesting that breakdown of superlubricity due to dynamic flake reorientation processes can be eliminated. Furthermore, at small contact sizes,  the rotated heterogeneous junction exhibits superlubric behavior up to considerably higher loads than the corresponding homogeneous junction. These results demonstrate that heterogeneous junctions of rigid layered materials are promising candidates for developing novel dry lubrication schemes that exhibit sustainable ultra-low friction and wear resistance.

## Methods

### Simulation setup

Figure 1 schematically presents our simulation setup aiming to mimic friction force microscopy (FFM) apparatuses commonly used in experiments. Here, a hydrogen terminated zigzag hexagonal graphene flake (represented, for simplicity, by a benzene molecule in the figure) is dragged along a rigid graphene or *h*-BN substrate. The pulling tip is represented by a rigid duplicate of the dragged flake positioned parallel to the underlying surface, whose center of mass is displaced along the substrate to represent a moving stage. Normal loads are modeled by applying a constant force at the center of mass of the rigid duplicate in a direction perpendicular to the substrate. In our simulations, the load values are varied in the range of 0.05–2.0 nN/atom corresponding to applied pressures of 2–80 GPa, typically investigated in experiments and simulations^13, 33^.

The interactions between the rigid duplicate and the sliding graphene flake are described by classical harmonic springs connecting each flake atom with its counterpart on the rigid duplicate. The presented results have been obtained for spring constants of ${K}_{||}=16\,meV/{\AA }^{2}$ in the lateral directions for all flake atoms and ${K}_{Z}^{C}=150\,{\rm{meV}}/{{\rm{\AA }}}^{2}\,\,$ and ${K}_{Z}^{H}=43\,{\rm{meV}}/{{\rm{\AA }}}^{2}$ in the normal direction for the carbon and hydrogen atoms, respectively. The former $({K}_{||})$ was previously used in ref. 13. for the homogeneous graphene interface and corresponds well with the equilibrium curvature of the Kolmogorov-Crespi^34^ (KC) potential for lateral displacements at the typically applied loads. The values of ${K}_{Z}^{C}$ and ${K}_{Z}^{H}$ are chosen to mimic the harmonic region of the KC potential and the graphene/*h*-BN interlayer potential (ILP)^25^ for vertical interlayer displacements near the equilibrium distance, respectively. We verify (see section S2 in the Supplementary Information) that our general conclusions are insensitive to the choice of these spring constants. Specifically, using infinitely rigid normal springs produced similar results to those presented above indicating that the main contribution comes from the adhesion forces that keep the slider close to the substrate.

The interlayer interactions between the sliding flake and the substrate are described by the KC potential and the graphene/*h*-BN ILP for graphene and *h*-BN substrates, respectively. For both substrates, the hydrogen/substrate interactions are adopted from the graphene/*h*-BN ILP. We note that these potentials neglect the effects of inter-layer covalent bonding that may occur in the presence of edge dangling bonds. In our simulations this effect, however, is expected to be of minor importance due to the chemical passivation of the edges of the slider. In realistic experimental scenarios a running-in procedure, where the interface is repeatedly sheared prior to the actual measurement until the friction-forces reach steady-state, can be utilized to effectively reduce the contribution of covalent interlayer bonding and surface contaminant effects.

The intralayer interactions within the dragged graphene flake are described using the well-established REBO^35^ potential. The corresponding equilibrium carbon-carbon distance is 1.3978 Å, yielding a lattice constant of 2.421 Å. This value is used to construct the underlying rigid graphene substrate whereas for *h*-BN we choose a lattice constant of 2.464 Å that approximately gives the experimental 1.8% intralayer lattice mismatch for the heterojunction. This methodology was shown to accurately reproduce the potential-energy surface for the two substrates considered^25, 36^.

We simulate flakes of different sizes including up to 7,000 atoms that corresponds to a contact area of ~180 nm^2^. We consider two different angular orientations of 0° and 30° between the crystallographic axis of the flake and the substrate (see Fig. 1). For the former, the starting configuration is chosen to be the optimal stacking mode of the flake on the corresponding underlying substrate. The starting configuration of the 30° misaligned system (see Fig. 1b) is obtained by a rotation of the aligned flake around its center-of-mass. In the Supplementary Information we report the coordinates of sample initial configurations for both orientations. Periodic boundary conditions are applied in the lateral directions and the substrate supercell is chosen to be sufficiently large to avoid interactions between periodic images of the sliding flake.

### Simulation protocol

The simulations are performed using the quasi-static protocol first proposed in ref. 37. While all atoms in the substrate are kept in fixed positions, at each step the image flake is rigidly displaced by 0.012 Å along the scan line shown in Fig. 1, and the driven graphene flake structure is relaxed until the forces acting on each degree of freedom reduces below a threshold of 3.12 × 10^−4^ eV/Å. Convergence of the results with respect to the tip displacement interval is verified. For structural optimization we adopted the FIRE algorithm^38^. The procedure is repeated for a global driven flake displacement corresponding to at least three periodicities of the underline substrate.

We note that this protocol is justified for low pulling velocities of 1–1,000 nm/s typically used in FFM experiments, and over-damped dynamics. In our simulations temperature effects are not considered. Previous studies^13^ on graphitic interfaces have shown that below a value of 10 K the frictional behavior of the system is weakly dependent on temperature. At higher temperatures, not modeled within the present study, friction is expected to reduce thus further supporting our main conclusions regarding robust superlubricity in the heterojunctions. The usage of a rigid substrate was previously validated by reproducing experimental results of a multi-layer graphene flake sliding atop graphite^13, 39^. Nevertheless, for thin substrates, in- and out-of plane elastic deformations may affect the measured friction, that becomes dependent on the number of substrate layers^40, 41^. In section S5 of the Supplementary Information we report results of a few tests performed considering an *h*-BN substrate bilayer, where the contacting layer was let free to relax. Kinetic and static friction are found to be only slightly larger compared to the simple rigid monolayer model, while the transition to smooth-sliding is not affected. A thorough analysis of all these effects, which we did not consider here, is currently underway.

Considering size dependence of the friction force, we performed simulations at normal loads up to a value of 0.2 nN/atom atom (see also section S1 in the Supplementary Information). For each size of the flake, the friction coefficients are extracted from the slope of the friction forces as a function of the external load, considering the initial Amontons-like linear regime observed at small loads. For the rotated junctions we have also studied the dependence of the friction force on the normal load doing simulations up to a value of 2 nN/atom. In this case, the friction coefficients have been evaluated considering the local, two-points derivative of the friction forces as a function of the load.

### Evaluation of the friction forces

The main observable in friction experiments is the lateral force acting on the tip. In our simulations this is represented by the lateral spring forces acting between the sliding flake and its rigid duplicate. Following an initial transient (that depends on the initial configuration), this force becomes a periodic function of the tip displacement, with a periodicity that equals the substrate lattice spacing. The static friction is evaluated as the maximum value of the lateral spring force obtained along the friction trace in the pulling direction. The kinetic friction is estimated as the lateral spring force averaged over a single period.

## Electronic supplementary material

Supplementary Information

Movie 1

Movie 2

Movie 3

Dataset 1

Dataset 2

Dataset 3

Dataset 4
