# Supplementary material for: Sliding friction of graphene/hexagonal –boron nitride heterojunctions: a route to robust superlubricity
Source: Sci Rep. 2017 Sep 7;7:10851. doi: 10.1038/s41598-017-10522-8 (PMC5589749; doi:10.1038/s41598-017-10522-8)
Supplement: Supplementary file 1 — Supplementary Information [file 41598_2017_10522_MOESM1_ESM.pdf]

Sliding friction of graphene/hexagonal-boron nitride heterojunctions: a route to robust superlubricity

Supplementary Information

D. Mandelli, I. Leven, O. Hod, and M. Urbakh

School of Chemistry and The Sackler Center for Computational Molecular and Materials Science, Tel Aviv University, Tel Aviv 6997801, Israel

S1. Load Dependence of Friction at the Aligned Configuration

In Figs. 2c and 2d of the main text, we have reported the static and kinetic friction forces dependence on the contact area, respectively, for the aligned homogeneous and heterogeneous junctions at an external normal load of 0.1 nN/atom. To demonstrate the general nature of our conclusions we repeat the calculations at external loads of 0.05 and 0.2 nN/atom. As can be seen in Fig. S1, a very similar frictional behavior is obtained at all external loads considered, thus verifying the broad range of validity of our results.

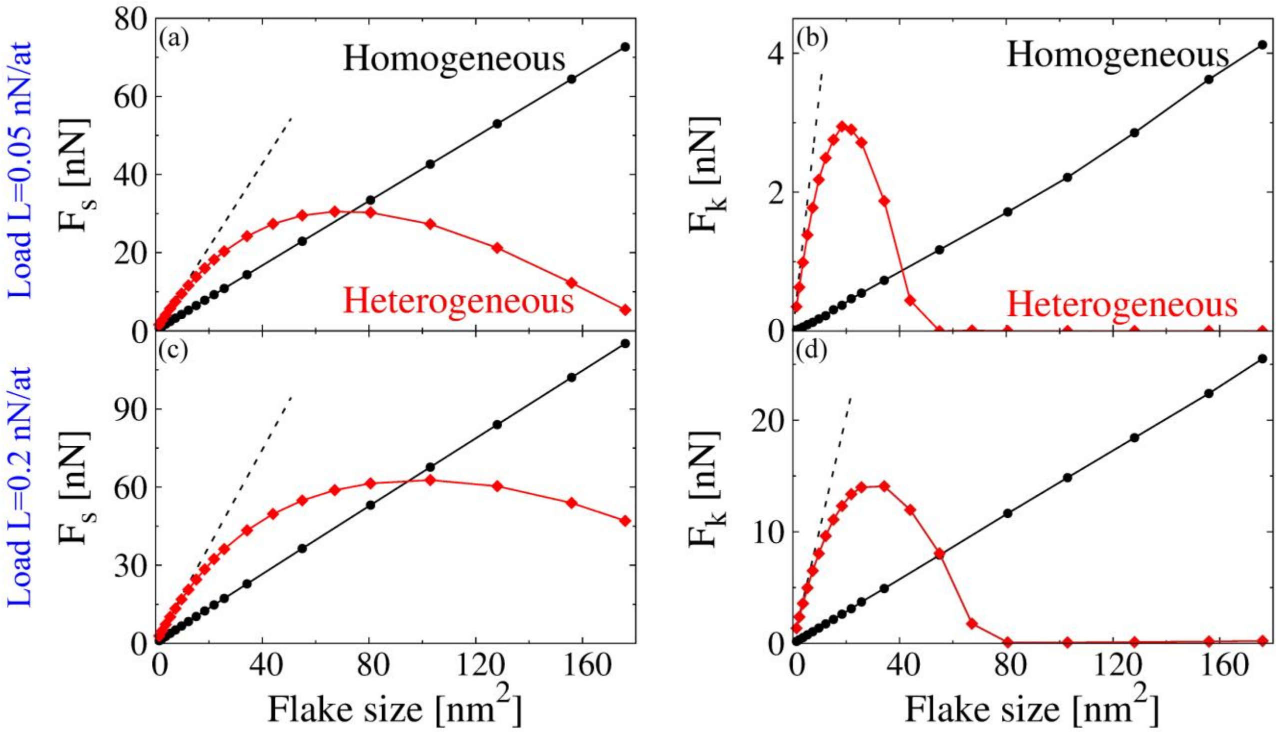

Supplementary Figure S1: Friction forces as function of contact size for the aligned (θ=0°) configuration at different external loads. Panels (**a**) and (**b**) present the static and kinetic friction forces, respectively, as a function of the contact size, calculated at an external load of L=0.05 nN/atom for the homogeneous graphene/graphene (black) and heterogeneous graphene/*h*-BN (red) contacts. Panels (**c**) and (**d**) present the corresponding friction forces calculated at an external load of L=0.2 nN/atom. In each panel the black dashed lines show the behavior of an artificially commensurate heterojunction having the lattice spacing of graphene. All results reported here were obtained with a lateral spring constant $K_{\parallel}$=16 meV/Å^2.

For completeness we report in panels (a), (b), and (c) of Figs. S2 and S3 the color maps of the intra- and interlayer distances, and of the local registry index,[1] respectively, corresponding to the starting configurations relaxed at a normal load of 0.05 nN/atom (Fig. S2) and 0.2 nN/atom (Fig. S3). Clearly, the area of the nearly commensurate domains (black regions in panels (c)), characterized by optimal

intralayer bond-length (white regions in panels (a)) and interlayer distance (blue areas in panels (b)), grows with increasing external load. This effect, along with the decrease of the interlayer distance, is responsible for the overall increase of friction as a function of the external load in the heterojunction. Nevertheless, a superlubric regime appears (see Fig. S1), even at the highest load considered, but with a larger onset with respect to contact size. The corresponding smooth soliton-like ridge propagation across the flake is presented in panels (d) of Figs. S2 and S3 for a contact size of 176 nm^2.

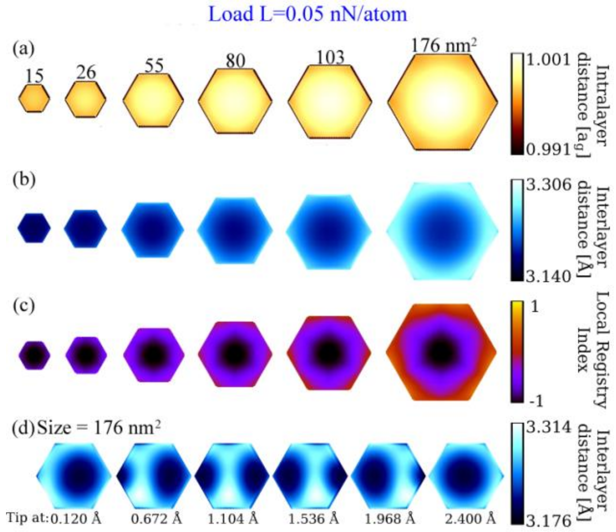

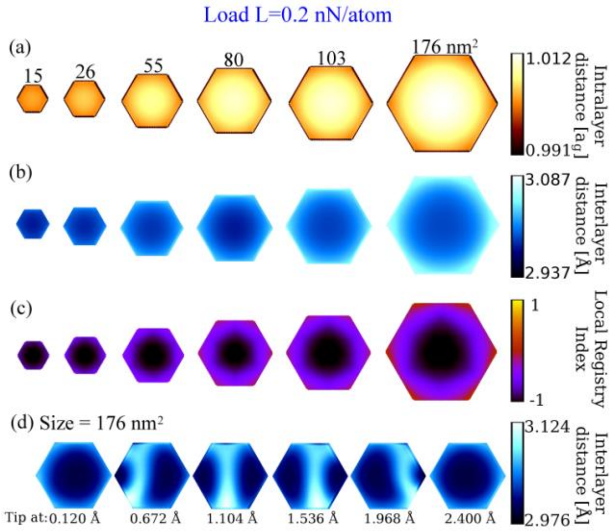

Supplementary Figures S2 (left) and S3 (right): Emergence of the Moiré superstructure with increasing graphene/*h*-BN heterojunction contact size at an external load of 0.05 nN/atom (left) and 0.2 nN/atom (right). Panels (**a**), (**b**), and (**c**) present the structures of hexagonal graphene flakes relaxed over a rigid *h*-BN substrate as function of contact size. The color maps of panels (**a**) and (**b**) show the normalized intralayer C-C bond length and the interlayer distance, respectively. Panels (**c**) report the local registry index maps, where dark regions correspond to the energetically favorable C stacking mode and bright regions correspond to the energetically unfavorable A stacking mode of the heterojunction, clearly indicating the appearance of the Moiré superstructure with increasing contact area. Panels (**d**) demonstrate the soliton propagation sliding mechanism during the lateral motion of the 176 nm^2 flake. All results reported in these figures have been obtained with a lateral spring constant $K_{\parallel}$=16 meV/$Å^2$.

S2. Static and Kinetic Friction: Intrinsic versus Extrinsic Nature

The frictional properties reported in the main text have been obtained using a lateral driving spring constant of $K_{\parallel}$=16 meV/Å^2 that corresponds to the equilibrium curvature of the Kolmogorov-Crespi[2] potential for lateral displacements at the typically applied loads. To evaluate the sensitivity of the static and kinetic friction forces towards this choice we compare, in Fig. S4, the contact size dependence of the friction coefficients and forces of the aligned (θ=0°) heterojunction obtained for lateral spring constants of 16, 30 and 40 meV/Å^2.

Static friction, which depends on the potential energy barriers for interlayer sliding, is an intrinsic property of the interface. Correspondingly, the static friction coefficient and force present the same contact size dependence regardless of the driving spring stiffness (see panels (a) and (c) of Fig. S4). On the contrary, while the qualitative behavior of the kinetic friction coefficient and force with contact size is similar for all driving spring constants considered, their magnitude strongly reduces with increasing spring stiffness. This can be rationalized by the simplified Prandtl-Tomlinson model showing that an increase of the driving spring constant, with respect to the stiffness of the sliding energy potential, results in reduction of kinetic friction. Furthermore, as $K_{\parallel}$ increases, the contact size (represented by N_{at}) required to satisfy the criterion for achieving superlubric motion ($\eta = 2\pi F_s/N_{at}K_{\parallel}a_{h-\mathrm{BN}} \approx 1$) reduces as is clearly demonstrated in panels (b) and (d) of Fig. S4.

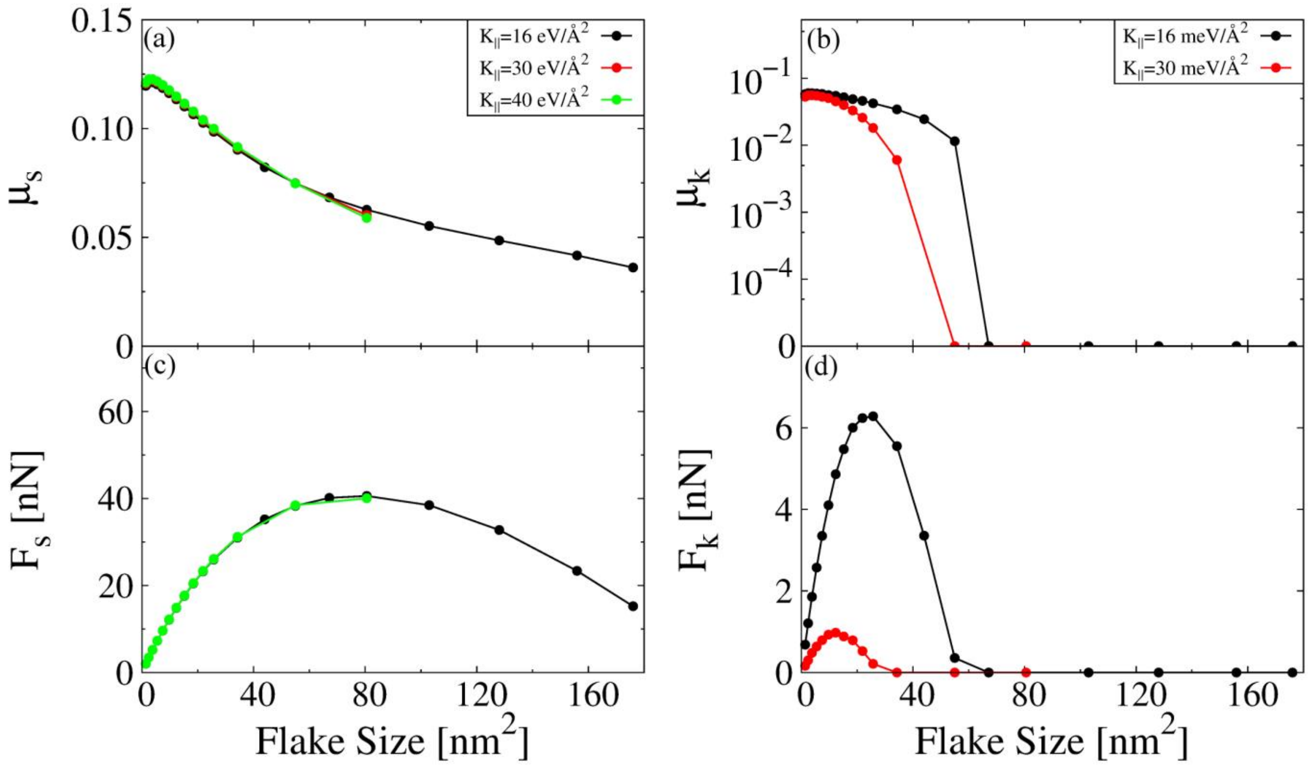

Supplementary Figure S4: Sensitivity of the static (left panels) and kinetic (right panels) friction coefficients (upper panels) and forces (lower panels) contact size dependence on the driving spring stiffness for the aligned ($\theta=0^o$) heterogeneous graphene/*h*-BN interface. The static friction results are presented for three driving spring constants of 16, 30, and 40 meV/Å^2. Panels (**c**) and (**d**) are data from simulations carried out at a normal load of 0.1 nN/atom. Short test calculations using $K_{\|}$=40 meV/Å^2 were performed only up to the first slip event, enough to calculate the static friction force and to check for the presence or absence of the stick-slip instability, but hindering the evaluation of kinetic friction.

For small flakes, where the friction trace exhibits periodic saw-tooth behavior, a simple relation between the static and kinetic friction forces can be derived that demonstrated their intrinsic and extrinsic characters, respectively. In Fig. S5 a typical friction force trace is presented for a flake size of 3.8 nm^2 under a normal load of 0.1 nN/atom driven via springs of lateral constant $K_{\|}$=16 meV/Å^2. Focusing on a single period, the triangular shape can be divided into two distinct sections: (i) a positive section representing the case where the flake is dragged by the tip; and (ii) a negative section, where the flake pushes the tip in the direction of sliding. In typical simulations, where the equilibrium spring length is chosen as zero, the latter represents an overshoot situation following the slip event, with the flake surpassing (and pulling) the tip in the sliding direction.

The static friction force $F_s(S, L)$, which depends on the contact size $S \propto N_{at}$ and the normal load L, is given by the peak of the force trace. This value is naturally set by the corrugation of the sliding potential energy surface that depends on the interlayer interactions and may vary with the flake size

and the normal load. The kinetic friction force is evaluated as the average force along one saw-tooth period. Assuming a perfect triangular shape the instantaneous friction force can be written as a linear function of the form $F_x = \frac{F_s - F_{min}}{a_{h-\mathrm{BN}}}(x - x_{min}) + F_{min}$, where x_{min} is the driving spring length yielding the minimal instantaneous friction force, F_{min}. Hence, the kinetic friction force can be calculated as:

$$F_k(S, L, K_{\parallel}) = \frac{1}{a_{h-\mathrm{BN}}} \int_{x_{min}}^{x_{min}+a_{h-\mathrm{BN}}} F_x dx = F_s(S, L) - a_{h-\mathrm{BN}}\, m(K_{\parallel}, S)/2, \quad \text{(S1)}$$

where $m(K_{\parallel}, S)$ is the slope of the force trace during the stick stage that represents the overall effective driving spring constant, which in turn can be approximated as the sum of all individual spring contributions $m(K_{\parallel}, S) \approx N_{at} K_{\parallel}$. Therefore, Eq. (S1) shows that the kinetic friction $F_k(S, L, K_{\parallel})$ is a decreasing function of $K_{\parallel}$.

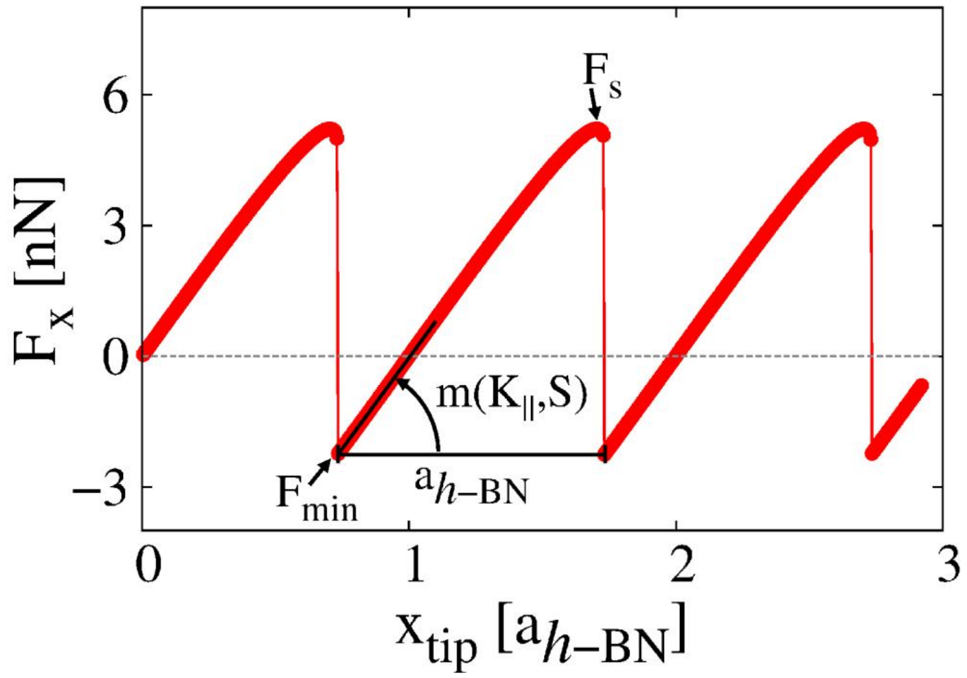

Supplementary Figure S5: Friction trace for the heterogeneous graphene/*h*-BN junction of contact size 3.8 nm^2 presenting typical saw-tooth behavior when dragged by a spring of lateral spring constant $K_{\parallel}$=16 meV/Å^2 under a normal load of 0.1 nN/atom. The static friction force (F_s), minimum friction force (F_{min}), substrate periodicity ($a_{h-\mathrm{BN}}$), and the linear force-trace slope (m) that define the saw-tooth curve are explicitly marked in the figure.

S3. Criterion for Transition from Stick-Slip Motion to Smooth Sliding

In the main text we demonstrated that the transition from stick-slip soliton motion to superlubric sliding can be characterized by a generalized Prandtl-Tomlinson (PT) parameter of the form $\eta = 2\pi F_s / N_{at} K_{\|} a_{h-\mathrm{BN}}$, where F_s is the size-dependent static friction force, N_{at} is the number of flake atoms, $K_{\|}$ is the lateral driving spring constant, and $a_{h-\mathrm{BN}}$ is the periodicity of the h-BN lattice along the scan-line. In the standard PT model the transition occurs at a value of $\eta = 1$. To evaluate the validity of this criterion in our fully atomistic simulations of the aligned ($\theta=0^o$) heterogeneous graphene/h-BN junction, we present in Fig. S6 the value of η as a function of flake size for several external loads and driving spring constants. For each curve, the transition point from stick-slip to smooth sliding is designated by a color change from black to red. Our simulations show that even when collective soliton motion dominates the frictional behavior of the system the onset of superlubricity occurs at a value of $\eta \approx 1$ regardless of the exact value of the external parameters. Furthermore, the transition from stick-slip to smooth-sliding occurs at contact sizes well below 200 nm^2 that roughly corresponds to a full Moiré superstructure periodicity.

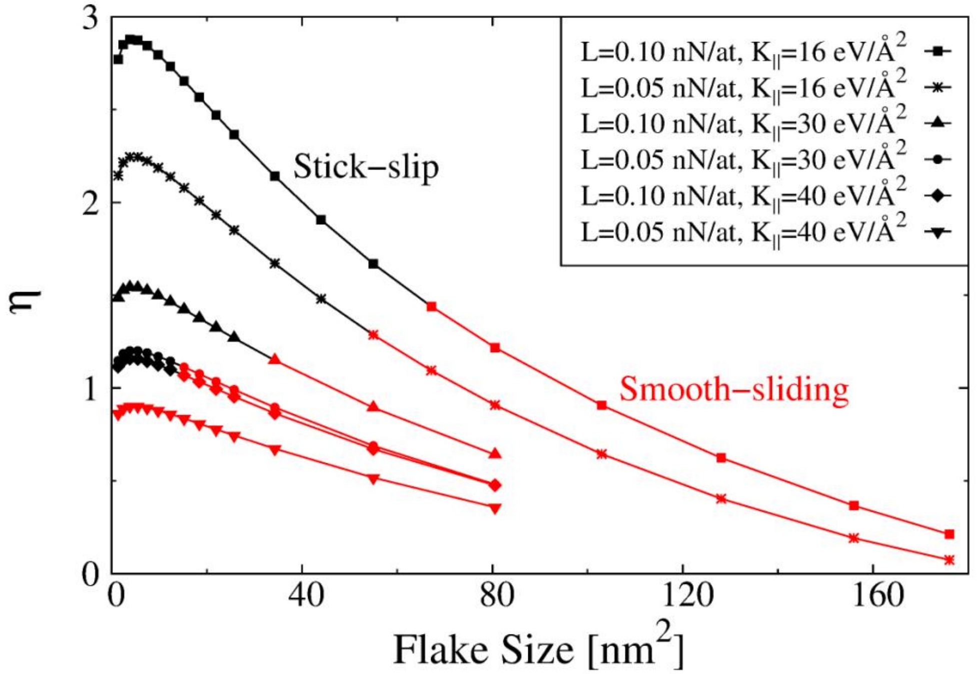

Supplementary Figure S6: The Prandtl-Tomlinson parameter, η (see text), of the aligned ($\theta=0^o$) graphene/h-BN heterogeneous interface as a function of contact size. Different curves correspond to different external loads of 0.05, 0.1 nN/atom (see main text) and lateral spring constants of 16, 30, 40 meV/$Å^2$. The latter correspond to a harmonic approximation of the ILP at the considered range of normal loads. Black branches correspond to simulations within the stick-slip regime, whereas red branches indicate the smooth-sliding regime.

S4. Independence of Friction on Contact Size in the Rotated Interfaces

In the main text it was noted that the friction exhibited by the rotated ($\theta=30^o$) junctions is found to be independent of contact size. To demonstrate this, we present in Fig. S7 the corresponding static friction coefficients (panel (a)) and forces (panel (b)) of the misaligned homogeneous (black) and heterogeneous (red) junctions as a function of the contact size. The friction coefficients are calculated as numerical derivatives of the friction forces with respect to the applied normal load in the interval of 0.05-0.2 nN/atom, well within the linear regime of friction force dependence on external load. The corresponding kinetic friction forces and friction coefficients of the rotated junctions are found to be below our numerical accuracy for all sizes and loads considered herein.

In the misaligned interfaces friction is dominated by edge effects. Specifically, the vertical position of the edge atoms is more susceptible to variations in the external load than that of the central flake atoms. As the normal load increases, the edge atoms approach the underlying substrate causing increased steric repulsions that result in higher sliding energy barriers and increased static friction. Hence, the edge contribution to the friction coefficients, defined as the variation of the friction force with external load, is more pronounced than that of the central flake atoms.

As can be seen in panel (a) of Fig. S7, for small flakes the static friction coefficients show strong dependence on the contact size, however as the edge to surface ratio decreases the edge effects become less dominant and the friction coefficients vary much less with contact size. In agreement with previous rigid nano-slider models,[3] we observe that the static friction force mildly fluctuates around a constant value as a function of the contact size (see Fig. S7b).

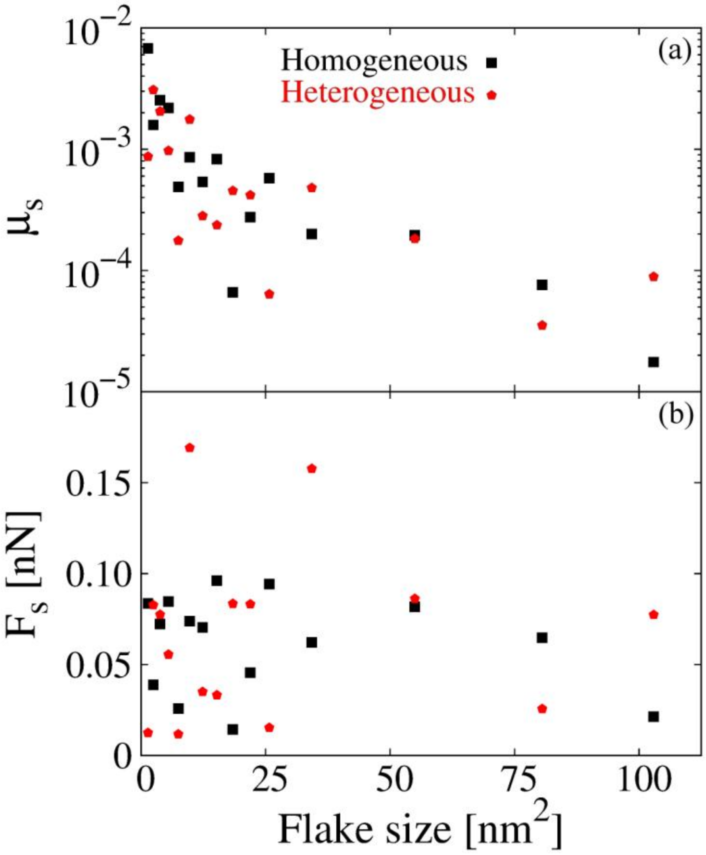

Supplementary Figure S7: Static friction coefficients (**a**) and forces (**b**) as a function of the contact size of the misaligned (θ=30°) homogeneous (black) and heterogeneous (red) junctions. Panel (**b**) reports data obtained at a normal load of 0.1 nN/atom. Similar trends were observed in the entire range of normal loads investigated (0.05-0.2 nN/atom). Simulations were performed adopting a lateral driving spring constant $K_{\parallel}$=16 meV/Å^2.

S5. Rigid versus Flexible Substrates

In the main text, we have considered a setup, where a flexible flake slides atop a fixed rigid substrate. To evaluate the effects of the rigid substrate assumption we present here results obtained for a flexible substrate modeled by a bilayer *h*-BN consisting of a flexible layer residing atop a fixed rigid layer support. Fig. S8 reports a comparison between the quasi-static friction traces of aligned ($\theta=0^o$) graphene/*h*-BN heterojunctions obtained using a rigid *h*-BN substrate monolayer (black curves) and a flexible substrate (red curves). As clearly seen, the friction force traces of the rigid and flexible substrate simulations are very similar for both flake sizes considered.

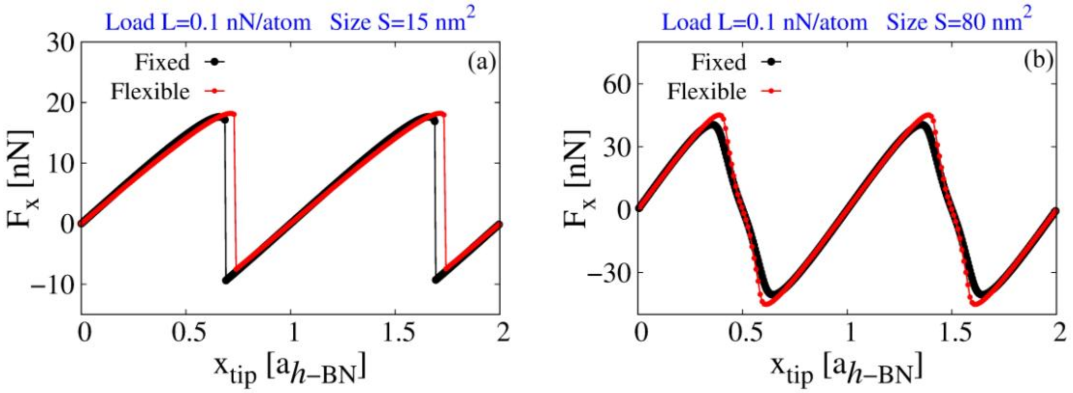

Supplementary Figure S8: Comparison of the friction force trace obtained for aligned ($\theta=0^o$) graphene/*h*-BN heterojunctions with contact areas of (**a**) 15 nm^2 and (**b**) 80 nm^2 using a fixed rigid monolayer substrate (black) and a bilayer substrate consisting of a flexible layer residing atop a fixed rigid layer support (red). All results reported herein have been obtained at a normal load of 0.1 nN/atom using a lateral spring constant value of 16 meV/Å^2.

Table S1 reports the numerical values of the static and kinetic friction forces extracted from the force traces of Fig. S8. The static and kinetic friction forces in the flexible substrate case are found to be somewhat larger (up to 15%) than for the fixed substrate case. Importantly, superlubric smooth sliding is obtained also in the flexible substrate case under the same condition as for the rigid substrate system.

	**Size = 15 nm^2**		**Size = 80 nm^2**	
**Substrate**	**F_s [nN]**	**F_k [nN]**	**F_s [nN]**	**F_k [nN]**
Rigid	17.6	5.5	40.6	0
Flexible	18.2	6.5	45.2	0

Supplementary Table S1: Comparison of the static and kinetic friction forces obtained for the aligned ($\theta=0^o$) heterogeneous junction with rigid and flexible substrate models at contact sizes of 15 and 80 nm^2. These simulations were performed at a normal load of 0.1 nN/atom using a lateral spring constant value of 16 meV/Å^2.

S6. Movies of the Sliding of Graphene Flakes on *h*-BN (Uploaded).

Movie1: Coherent stick-slip motion of a small flake of size S=2.4 nm^2 under a normal load of 0.1 nN/atom.

Movie2: Soliton-like stick-slip motion of the elevated Moiré ridge in an intermediate flake of size S=55 nm^2 under a normal load of 0.1 nN/atom.

Movie3: Soliton-like smooth sliding of the elevated Moiré ridge in a large flake of size S=176 nm^2 under a normal load of 0.1 nN/atom.

S7. Coordinate Files Including Several Initial Configurations for Homogeneous and Heterogeneous Junctions (Uploaded).

The uploaded material includes four XYZ-files with the configurations of aligned and rotated contacts of the flake of size S=80 nm^2 on graphene and *h*-BN. In each file, the edges of the 2D rectangular supercell are reported in the comment line.

REFERENCES

(1) Leven, I., Guerra, R., Vanossi, A., Tosatti, E., Hod, O. Multiwalled nanotube faceting unravelled. *Nat. Nanotechnol.* **11,** 1082 (2016).

(2) Kolmogorov, A. N., Crespi, V. H. Registry-dependent interlayer potential for graphitic systems. *Phys. Rev. B* **71,** 235415 (2005).

(3) De Wijn, A. S. (In)commensurability, scaling, and multiplicity of friction in nanocrystals and application to gold nanocrystals on graphite. *Phys. Rev. B* **86,** 085429 (2012).
